# Qualitative Evaluation of Advanced Care Planning in Early Dementia (ACP-ED)

**DOI:** 10.1371/journal.pone.0060412

**Published:** 2013-04-10

**Authors:** Michaela Poppe, Sarah Burleigh, Sube Banerjee

**Affiliations:** 1 Section of Mental Health and Ageing, Health Services and Population Research Department, Institute of Psychiatry, King's College London, London, United Kingdom; 2 Nursing Directorate, South London and Maudsley NHS Foundation Trust, Maudsley Hospital, London, United Kingdom; 3 Brighton and Sussex Medical School, University of Sussex, Brighton, East Sussex, United Kingdom; "Mario Negri" Institute for Pharmacological Research, Italy

## Abstract

**Background:**

End-of-life-care is often poor in individuals with dementia. Advanced care planning (ACP) has the potential to improve end-of-life care in dementia. Commonly ACP is completed in the last six months of life but in dementia there may be problems with this as decision-making capacity and ability to communicate necessarily decrease as the disease progresses. Choosing the right time to discuss ACP with people with dementia may be challenging given the duration of the illness may be up to nine years.

**Aims:**

To explore the acceptability of discussing ACP with people with memory problems and mild dementia shortly after diagnosis.

**Methods:**

In-depth interviews were conducted with 12 patients and eight carers who had participated in ACP discussions and six staff members from a memory clinic and a community mental health team who had either conducted or attended the discussions for training purposes.

**Results:**

Patients and carers found ACP a positive intervention that helped them think about the future, enabled people with dementia to make their wishes known, and resulted in their feeling relieved and less worried about the future. The importance of sharing the ACP documentation between health service providers was highlighted.

**Conclusions:**

This qualitative evaluation of ACP in early dementia has encouragingly positive results which support the wider application of the intervention in memory services and community mental health teams. Strategies are suggested to support the implementation of ACP further in clinical practice.

## Introduction

Dementia is a devastating illness that affects cognitive, behavioural and physical functioning. The number of people with dementia worldwide is estimated to be 36 million. This number is projected to rise to 115 million by 2050 [1]. Research has shown that the quality of end of life care in people with dementia is often poor [2]. The Department of Health for England developed their End of Life Care Strategy to “improve the provision of care for all adults at the end of life, and their family and carers” [3]. Advance care planning (ACP) is a core element of that programme. ACP refers to a process of discussing an individual's preferences for care they would like to receive at a time when they may no longer be able to make such decisions or their wishes known.

While most of the general public (60–90%) supports the idea of ACP, only 8% of individuals in England and Wales have completed ACP documentation in comparison with 10–20% of individuals in the US, Canada, Australia, Germany and Japan [4], [5], [6]. Little research has been conducted on ACP in dementia. Previous research on non-demented populations suggests that earlier discussions may be associated with an increase in feelings of autonomy [7], maintenance of control, patient satisfaction [8], [9] and improved quality of care and reduced stress, anxiety and depression in family members [10].

Where there is no advance care plan to provide information about a patient's preference for care, and the patient cannot make their wishes known, their next of kin tends to be asked to make the decisions about what end of life care would best reflect the patient's wishes. However, in one third of cases, patient-proxy decisions may be inconsistent [11]. Also carers find making decisions on behalf of the patient difficult, especially around areas such as care homes, legal matters and end of life care [12].

Commonly ACP is completed in the last six months of life, in dementia there may be problems with this as decision-making capacity and ability to communicate necessarily decrease as the disease progresses. Choosing the right time to discuss ACP with people with dementia may be challenging given the duration of the illness may be up to nine years [5]. The National Institute for Clinical Excellence and Social Care Institute for Excellence [6] suggest that ACP should be discussed while the individual still has mental capacity to make decisions but ACP has not yet been integrated into routine practice in dementia [13]. One reason for this is that there have been concerns that raising issues of end of life care early in dementia might be difficult and unacceptable to people with dementia and family carers. This study was designed to evaluate the acceptability of a systematic dementia-specific approach to ACP discussion.

## Methods

### Ethics statement

All participants gave their informed written consent. Capacity was a prerequisite for the ACP discussion with people with dementia in this study, so all patients interviewed about their experiences of the ACP discussion had capacity to consent. The study was approved by the South East London REC 3 Research Ethics Committee.

### The Advanced Care Planning in Early Dementia tool (ACP-ED)

ACP tools have been developed to structure discussions about end of life care and to structure records of these discussions [14], [15], but there are none that have been developed for use in early dementia. We therefore developed the Advanced Care Planning in Early Dementia tool (ACP-ED). An initial draft was devised and then iteratively revised following discussion with people with dementia, carers, and dementia practitioners. This was completed at patient and public engagement groups, conferences, and patient and carer participation events organised by the South London and Maudsley NHS Trust. In this opinions were gathered and tested about what should be covered, which questions to use, and style and language. Overall, 18 patients, 25 carers and 150 members of staff provided feedback in the development phase of ACP-ED. The ACP-ED is presented in Figure S1.

### ACP discussions

ACP discussions were conducted with patients from two memory services in south London. The memory services identified patients for ACP discussions either during the diagnostic assessment or from their case load of cases with mild dementia. ACP discussions were conducted by a senior nurse (SBu) and by a clinical psychologist. For training purposes the specialist nurse who conducted the discussions invited members of the memory service to observe the ACP discussions (with the patient's and carer's consent). This was because a sustainable model for uptake would require that ACP discussions should be conducted and reviewed by memory service staff as an extension of their current role in the future.

### Evaluation of ACP discussions and the ACP-ED tool

#### Participants

In-depth individual qualitative interviews were conducted with three groups of participants: people with mild dementia, carers of people with mild dementia and staff from a memory service and a community mental health team for older people. Patients and carers who had taken part in an ACP discussion using the ACP-ED were asked if they would agree to be contacted by a researcher to discuss the possibility of being interviewed about their experience of the ACP discussion. Patients and carers who agreed were sent an invitation letter containing an information sheet about the interview. Patients and carers were subsequently contacted by telephone to discuss participation. In addition, the nurse and the clinical psychologist who conducted ACP discussions as well as four team members who attended an ACP discussion were interviewed.

#### Data collection

Interview guides were developed based on the research literature. Questions were open-ended and revised iteratively to further explore issues raised. Interviews with patients and carers explored issues around diagnosis, what prompted them to discuss ACP, and an evaluation of the ACP discussion. Staff interviews covered the ACP-ED tool, the ACP discussion, barriers and facilitators to conducting ACP as well as skills and competencies required for discussing ACP. Interviews with patients, carers and staff were conducted by a researcher with extensive experience in dementia research (MP). Interviews lasted an average of 45 minutes. Interviews with patients and carers were conducted in the patients' homes; interviews with staff were conducted at their place of work.

### Data Analysis

Interviews were audio-recorded and transcribed verbatim. Data from the interviews were separated into meaningful fragments and emerging themes were labelled with codes. The constant comparison method [16] was used to identify similarities and differences between emerging themes. Interviews with the three different groups of participants served as a means of triangulation to allow for a more comprehensive understanding of the topic [17]. MP and a senior qualitative researcher independently coded the initial transcripts and compared coding strategies. Disagreements between the raters were resolved by discussion. A coding book was developed and applied to the remaining transcripts by MP. NVivo 8 software [18] was used to aid the analysis of the interviews.

## Results

ACP discussions were held with 16 people with dementia, 14 agreed to be approached for the evaluation. Of these, 12 people with dementia and eight carers consented to be interviewed about their experience of the ACP discussion. The main themes that emerged from the interviews were: motivation for ACP, views of the ACP discussion and timing of the discussion. The main themes emerging from staff interviews were: challenging aspects of ACP, views of the ACP-ED tool, timing of the discussion, barriers and facilitators of ACP and skills and competencies. These will be discussed below, quotations are labelled as P for people with dementia, C for carers and S for staff. See Table S1 for characteristics of participants.

### Motivation for ACP

Only one third of the patients interviewed had thought about any aspect of the future prior to the ACP discussion. On being offered the chance motivation to agree was divided between concern about their memory and wanting to plan for a time when they could no longer look after themselves. One patient wanted to discuss preferences for the future because of a dispute with a family member, who was questioning the patient's capacity to make decisions. Having made his preferences for future care known, he felt more secure and considered the plan as a means of self-protection.

### Views of the ACP discussion

All but three patients considered ACP a positive and helpful experience and were satisfied with having had the discussion.

*‘I was glad to have told her what I wanted.’ (P8).*

*‘They covered everything I wanted to know and the questions they asked were the right questions.’(P3).*

Patients said that the ACP discussion gave them time to think about the future. Some stated that they were relieved and less worried after discussing their preferences for the future. They felt reassured about the support from their family and services and they found it important that their family and professionals knew their preferences for the future.

*‘I suppose really it was the wisest thing to do because there is no use leaving things like that too long before things are going to get worse. You don’t know what you are doing. I would rather know what I am doing so that's why I decided to make arrangements and things so if anything happens now they all know, both of them know, what I want and what's happening and so it saves me worrying about it.’ (P12).*

On the negative side, two patients found discussing the future dispiriting, while another found discussing the future difficult without knowing what the future would bring.

All carers agreed that ACP was a positive experience. Carers said that ACP made them think about the future and that the initial ACP discussion prompted further discussions about the future with the patient or other family members. Two carers mentioned they had tried to discuss the future with the patient before and had found it difficult. They felt they probably would not have brought up the topic again without the ACP discussion being prompted by the memory service. Carers liked that ACP gave patients the opportunity to express preferences for their care; they considered it helpful to find out the patient's wishes and to have a written record of it, so that everyone knew that this was what the patient wanted. Carers expressed relief that they had discussed the future with the patient. They felt more confident that if necessary they would be able to make a decision that would reflect the patient's wishes.

*‘The social worker doesn't know mum and doesn't know us and whereas we are actually quite a close knit family and we are very lucky because we can actually make those decisions and think yeah that isn't actually what mum would want, what she would want is x, y, z.’ (C12).*

The need for regular review of any ACP document was identified in case patients' preferences changed. One carer stressed the importance of communicating the ACP documentation to other relevant health service providers with the patient's consent and suggested that service providers should receive training in order to understand ACP and the relevant ACP documentation. While most patients and carers said they would recommend ACP and were strongly support of it being offered, they added that ACP should only be discussed if it was the person's choice and if they were ready for the discussion.

### Challenging aspects of ACP

Staff considered end of life care the most challenging aspect of the ACP discussion because they felt the topic could cause some anxiety in patients. Staff had particular concerns with the subject of assisted suicide being raised by patients. But it was striking that this was not brought up by patients or carers.

One staff member discussed an ACP discussion that had gone well until the subject of end of life care was broached. The carers were upset by the topic and the staff member wondered whether it was too early in the course of the patient's illness to discuss end of life care. However, she felt that it was important to discuss the topic while the patient was still able to make such decisions because the family might not necessarily share the patient's views about end of life care.

Staff reported that patients frequently asked to be given a timescale for dementia progression. However, given the heterogeneity of dementia, staff found it difficult to discuss the disease trajectory in their individual case. They felt that this uncertainty about the duration of the illness made it difficult for patients to plan for the future.

Patients' lack of understanding of dementia was also cited as a difficult aspect of the ACP discussion, with one staff member indicating that some patients' decisions may not have been as informed as they perhaps could have been. Discussing the patient's living situation was considered as a potentially difficult topic because patients might find the thought of having to potentially leave their home to go into a care home distressing.

### Assessment of the ACP-ED tool

Staff, patients and carers believed that all relevant issues were covered in the ACP-ED tool. Staff found it useful that the tool provided structure to guide them in the discussion. They thought it was helpful that the tool was open-ended, as it provided flexibility and the given questions could generate further questions. It was possible that the open-endedness could also be a disadvantage if a patient was vague. Staff who had not yet conducted any advance care planning discussions themselves were unsure how to initiate the discussion with those patients who had not raised the issue themselves, but saw the tool as a potential way of facilitating this.

### Timing of the discussion

Patients, carers and staff agreed that ACP should be discussed sooner rather than later. Staff found it difficult to pinpoint a specific time in the dementia pathway for discussing ACP, but the general consensus was that the opportunity to discuss ACP should be offered to patients soon after diagnosis when patients had time to think about the diagnosis, when they were still in contact with the service, and where they were still able to make decisions about preferences for the future. There was overall agreement between staff that doing this at the point of diagnosis might be too stressful.

*‘It's very difficult because when is the best time? I often think probably when you are feeding back or once you just had the diagnosis, had time to digest it a bit, consider what that might mean to them and then maybe a month after that or something, that might be a good time, not before because it doesn't mean anything to them and not during because it's too overwhelming I think.’ (S3).*

Some patients and carers stressed that the timing of the discussion should depend on individual circumstances and whether they were ready to discuss ACP. They suggested memory services could advise on the right time of the discussion based on the results of their assessments and their experience with dementia progression.

### Barriers and facilitators to ACP

Staff thought that the main barrier to ACP on the part of the patients and carers was difficulty in some patients or carers to accept the diagnosis. One staff member cited the example of a colleague who found discussing ACP with a patient and carer problematic because some family members were disputing the diagnosis. Others said that some patients were worried that by discussing advance care planning, they would no longer be allowed to make decisions. They stressed the importance of giving patients and carers detailed information about ACP before the discussion took place, so that patients would not feel threatened by the discussion and so they could decide whether to proceed. Another potential barrier was whether patients were ready to discuss advance care planning. It was stressed by all that time was needed to come to terms with diagnosis before being able to start thinking about the future. Family dynamics was another potential barrier, one staff member gave an example of a case where the patient would have agreed to ACP, but the carer was against it.

*‘I think the client would have been quite open to the discussion but the daughter was quite, that wasn't somewhere that she wanted to do and she was, so we didn't.’ (S2).*

Lack of capacity was identified as another barrier to discussing the future with patients and introducing the topic of ACP early in the dementia pathway was seen as the solution. Staff thought that the main potential barrier on the side of staff was a lack of confidence in discussing ACP. The training package and the ACP-ED tool were seen as ways of addressing this. Staff were concerned that discussing ACP might be time consuming. They thought in some cases the discussions might require more than one session and advance care plans would have to be reviewed. This would need to be recognised as a core part of the service offered to those diagnosed with dementia.

Having built a good relationship with the patient and the patient's family was seen as a facilitator for advance care planning by staff members. Staff felt a patient would be more open to discuss ACP if they knew and trusted the person delivering the intervention. Moreover, they thought good training and refreshers made staff feel more confident about ACP, and good preparation was seen as important for facilitating the discussion.

### Skills and competencies

Staff identified knowledge about dementia, knowledge about available resources and knowledge of one's own limitations as key skills and competencies for discussing ACP. Staff stressed it was important to feel confident when discussing ACP and they found having experience in dealing with difficult conversations increased their confidence.

*‘I think it does draw on quite a complex set of clinical skills as well in terms of having difficult conversations and knowing that actually it's OK to push these conversations and not to back off these conversations. I think if I had done this sort of thing as a trainee, I would have backed off the conversation immediately and probably brought it to a premature close whereas I think because I've had a bit more experience, I persisted with the conversation even though it's upsetting and difficult.’ (S5).*

Staff said it was important to be perceptive about how the patient felt during the discussion, to conduct the discussion in a sensitive way and to be able to listen and let the patient guide the discussion as much as possible. They highlighted the importance of being open minded and not judging patients for their wishes. Good communication skills were another key competency that was identified by staff as well as the ability to manage conflict.

## Discussion

### Advance care planning in dementia is a positive intervention

The evaluation suggests that the ACP-ED tool can, with training enable advanced care planning in people with mild dementia following diagnosis. It provides evidence that such an approach can be acceptable and perceived as a positive and useful intervention that they would recommend to other people by people with dementia and their family carers. Carers found it helpful to know the patient's wishes in case they had to make a decision on behalf of the patient in the future.

But choosing the time to carry out ACP is important. This evaluation suggests that the best time to discuss ACP is soon after diagnosis when patients have had time to think about the diagnosis and the future but still have the capacity to make decisions about future care. This is in line with other studies in older people and dementia [19], [20], [21].

### The ACP-ED in clinical practice

This study demonstrates the feasibility of the intervention in people with early dementia and their cares in memory services. However two issues seem to be vital if this is to be successfully implemented for the benefit of all. First It is crucial that the topic is initiated by staff because patients and carers are unlikely to initiate the discussion with professionals spontaneously [22]. Second, services need to see this as a core part of their work and part of providing a good diagnostic service.

One of the main reasons why advance care planning has not been more widely implemented in practice is a lack of clarity about who should be delivering the intervention. Should ACP be initiated in primary care or within memory services? Our findings suggest that patients, carers and staff believe that memory services and CMHTs are well placed to initiate advance care planning discussions with individuals with dementia, provided they are properly trained and resourced. Staff identified training and supervision as key factors to increasing their confidence in initiating advance care planning discussions. Therefore, it is crucial to provide ACP training to staff who will be conducting the discussions and to offer supervision. It is positive that this function has been specified as a part of memory assessment services in the Commissioning Guidance issued by the Department of Health in England.

Concerns were raised by carers and staff about communicating patients' wishes to other health service providers. To enable implementation of the patient's wishes, it is important that the ACP documentation is made available to the relevant health service providers such as GPs with the patient's consent. Taken together these actions would enable people with dementia not only to live well with dementia, the title of the National Dementia Strategy for England, but also, vitally, to die well with dementia [23].

### Strengths and limitations

Patients, carers and staff from two memory services were interviewed. There is the possibility that other services in other areas might have had different responses to the raining and to the ACP-ED tool. Further quantitative evaluation is needed to determine whether there are benefits to patients and carers in the short and long term. We must be cautious with regard to the findings of our evaluation due to the small sample size and no conclusions can be drawn about the frequency of attitudes and reactions observed. However we did use good quality qualitative methodology and the views expressed are accurate representations of those of the patients, carers and staff interviewed.

## Supporting Information

Figure S1 **ACP-ED Tool.**
(DOC)

Table S1 **Characteristics of participants.**
(DOCX)
