# Supplementary material for: Qualitative Evaluation of Advanced Care Planning in Early Dementia (ACP-ED)
Source: PLoS One. 2013 Apr 10;8(4):e60412. doi: 10.1371/journal.pone.0060412 (PMC3629937; doi:10.1371/journal.pone.0060412)
Supplement: Figure S1 — ACP-ED Tool. (DOC) [file pone.0060412.s001.doc]

**
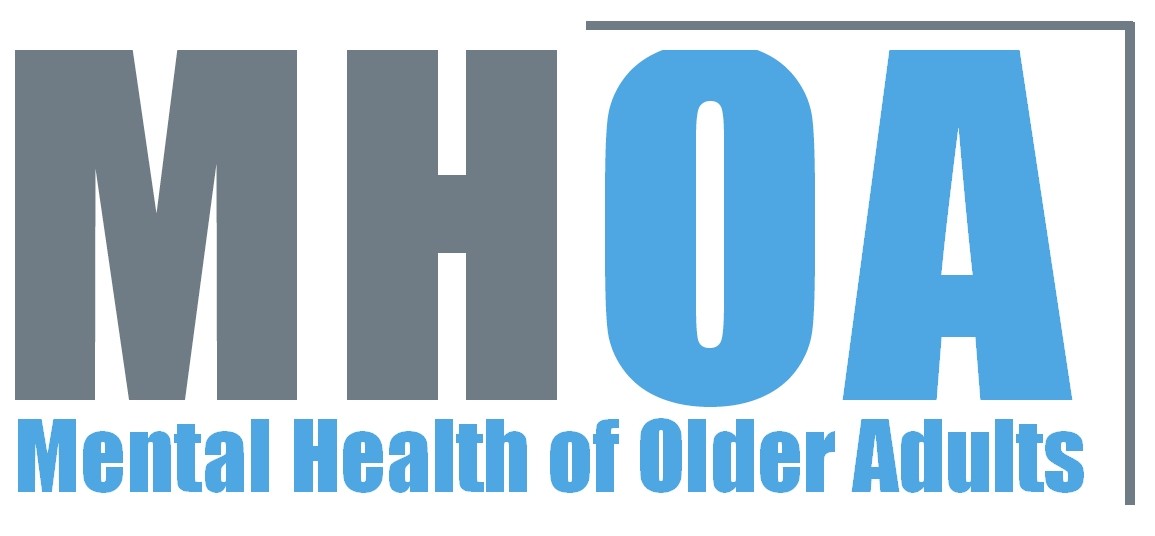
**

**ACP-ED Preferred Priorities for care**

**Planning for the Future**

**Name:-**

**Date of discussion :-**

This is an opportunity for you to have a discussion with your care team about your future care. It might help in your preparation for the future, and it is an opportunity for you to talk about and write down your priorities, and preferences for your care if and when your condition changes.

It is something that you can participate in, if you want to. If you would rather not discuss your future care at this point, your decision will be respected. The team will give further opportunities for this discussion to happen again in the future.

If a time comes when, for whatever reason, you are unable to make a decision yourself, anyone who has to make decisions about your care on your behalf will have to take into account everything that you have included in this plan.

Your views may change over time, and this plan can be changed or added to at any point. It will be regulalry reviewed by the team.

The professional team are there to help support you and the significant people in your life through this process. It can often be a very useful process to talk through your specific needs. It can also help you, your family and friends if they are going to be involved with your future care.

Some of the things that you might want to consider including in your plan, are your beliefs and values, what is important to you, what you would and would not like to happen, and where you would like to be cared for at the time in your life when your condition means that you need extra care and support.

You might have formally appointed someone to make decisions on your behalf, ( Lasting Power of Attorney) if so, please provide their details below:-

**These are some potential questions which might help the patient and their carer/family focus on the discussion.**

*How have you been feeling since you were given your diagnosis?*

MM

*What is your current understanding about the changes that might happen to you due to your condition over time?*

Mr Durban had a realistic approach to his diagnosis of dementia, and did speak about his feelings of frustration when he can not remember things. Plans are currently being discussed with his son and family about spending more time in somerset with his son and his family.

*What would you like to know about your care and treatment, how much information do you normally like to have? Are you the sort of person that likes to have all of the information, or would you prefer not to know too much?*

*Have you had any thoughts, discussions with your family or friends about what you would like to happen, if you become very ill, and needed more support and care.*

*Do you have any specific religious or spiritual needs which you would like to be adhered to wherever you are cared for, such as attending a local church, or meeting place?*

*Do you have any specific cultural needs that people need to be aware of in relation to your care, or any specific dietary preferences such as being a vegetarian?*

*If you became physically unwell, or if the changes that were happening to you became difficult to manage at home, where would you like to be cared for; Residential care, Home, Hospice, Nursing Home?*

*Would you like other people to be involved in your care, family, friends, significant others, professional carers?*

*Have you got any concerns that have not been discussed or addressed within this document?*

Signature of patient:-

Date:-

Staff member Signature:-

Date for Review:-
