# Supplementary material for: Qualitative Evaluation of Advanced Care Planning in Early Dementia (ACP-ED)
Source: PLoS One. 2013 Apr 10;8(4):e60412. doi: 10.1371/journal.pone.0060412 (PMC3629937; doi:10.1371/journal.pone.0060412)
Supplement: Table S1 — Characteristics of participants. (DOCX) [file pone.0060412.s002.docx]

**Table S1 Characteristics of participants**

| **Characteristic** | **Value (%)** |
| --- | --- |
| ***People with dementia* (n=12)** |  |
| **Age** | 78.75 ± 6.17, Range 68-88 |
| **Gende**r |  |
| Male | 4 (33.3%) |
| Female | 8 (66.7%) |
| **Living situation** |  |
| Living alone | 6 (50.0%) |
| Living with spouse/partner | 5 (41.7%) |
| Care home | 1 (8.3%) |
| **Marital status** |  |
| Single | 2 (16.7%) |
| Married | 4 (33.3%) |
| Divorced | 1 (8.3%) |
| Widowed | 5 (41.7%) |
| ***Carers* (n=8)** |  |
| **Gender** |  |
| Male | 4 (50%) |
| Female | 4 (50%) |
| **Relationship to person with dementia** |  |
| Spouse/ partner | 3 (37.5%) |
| Son/ daughter | 4 (50.0%) |
| Son/ daughter-in-law | 1 (12.5%) |
| ***Staff* (n=6**) |  |
| Job title |  |
| Community Practitioner | 3 (50.0%) |
| Team Manager | 1 (16.7%) |
| Clinical Psychologist | 1 (16.7%) |
| Assistant Director of Nursing | 1 (16.7%) |
| **Place of work** |  |
| Memory Service | 4 (66.7%) |
| Community Mental Health Team | 1 (16.7%) |
| Nursing Directorate | 1 (16.7%) |
